# First person – Yeon Ja Choi

**DOI:** 10.1242/dmm.052680

**Published:** 2025-11-21

## Abstract

First Person is a series of interviews with the first authors of a selection of papers published in Disease Models & Mechanisms, helping researchers promote themselves alongside their papers. Yeon Ja Choi is first author on ‘ Identification of conserved residues essential for the ciliogenic functions of WDPCP’, published in DMM. Yeon Ja conducted the research described in this article while a postdoctoral associate in Jiang Chen's lab at Stony Brook University, Stony Brook, NY, USA. She is now an associate professor at Jeju National University, Jeju, Republic of Korea, investigating the functions of ciliary proteins and their relevance to disease.


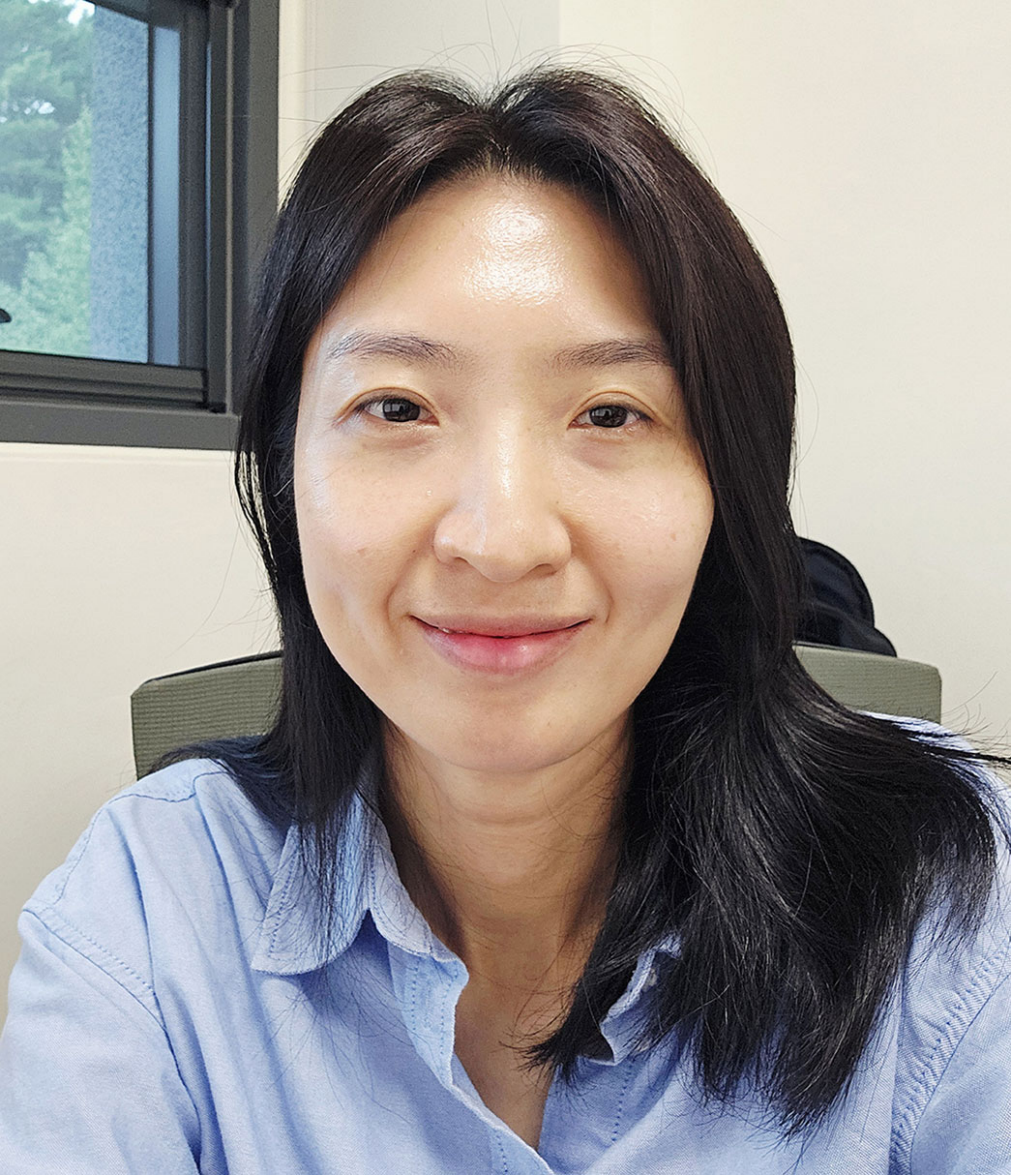


**Yeon Ja Choi**

**Who or what inspired you to become a scientist?**

I vividly remember the moment in elementary school when I first observed onion root tip cells dividing under a microscope. Watching the precise and logical process happened inside the cell was fascinating and made me full of wonder. That early experience sparked my interest in science, especially biology. Later, I decided to go to pharmacy school because I wanted to become a pharmacist to offer direct help to patients. Close to graduation, I realized that I was drawn to basic research and the fundamental questions of biology and disease. This realization led me to graduate school and the path toward becoming a pharmaceutical researcher. During my undergraduate years, I was deeply impressed by the passion my professors had for their research. The graphs and images in their lectures and conference posters looked like art works to me. That impression inspired me to contribute to that world of basic research. Together, these experiences shaped my professional journey and continue to guide my scientific career.

**What is the main question or challenge in disease biology you are addressing in this paper? How did you go about investigating your question or challenge?**

The main question we addressed was how the structure of WDPCP relates to its function in ciliogenesis and disease. Compared to other CPLANE proteins (i.e. INTU and FUZ), the biological function of WDPCP was just as crucial. But its structure-function relationship was poorly defined. WDPCP was named after its WD domains. At the time when we began this investigation, only two WD repeats had been predicted. Now, seven WD repeats have been identified. A number of patient mutations had been identified outside the WD domains, suggesting that the unexplored regions outside the WD repeats could be functionally important.

To investigate, we created a genetically engineered mouse model carrying a two-codon deletion (D481 and W482) in a conserved but previously uncharacterized region of WDPCP. We also used AlphaFold2 structure prediction and molecular dynamics simulations to examine how this deletion affected protein stability. By combining structural modeling with *in vivo* and *in vitro* experiments, we provided new insight into how conserved residues affect WDPCP protein structure and maintain WDPCP function.

We discovered that removing just two tiny pieces of the WDPCP protein was enough to stop cilia from forming and to block key cell signaling pathways

**How would you explain the main findings of your paper to non-scientific family and friends?**

Our study focused on a protein called WDPCP, which is important for making cilia. Cilia are tiny hair-like structures on the surface of cells that act like antennae, helping cells sense signals and communicate with their surroundings. When cilia do not form or function properly, it can cause serious developmental problems and rare genetic diseases called ciliopathies. We discovered that removing just two tiny pieces of the WDPCP protein was enough to stop cilia from forming and to block key cell signaling pathways. Losing just these two small pieces had the same effect as losing the entire protein, and this region had not previously been reported. This shows how critical those tiny pieces are for the function of WDPCP and helps explain why mutations in this region can lead to disease.

**What are the potential implications of these results for disease biology and the possible impact on patients?**

The residues we identified represent a mutation in a region of WDPCP that had not previously received much attention. Unlike many other mutations located within the WD repeat domains that form the beta propeller, or those that cause unstable transcripts and reduce protein levels, this mutation lies outside the beta propeller in the C-terminal region. Our findings highlight the functional importance of this previously overlooked part of the protein. Importantly, a mutation at the same tryptophan residue in humans has been reported in ClinVar, and our study provides a mechanistic explanation of how such a mutation can contribute to the development of Bardet-Biedl syndrome (BBS).

**Figure:**
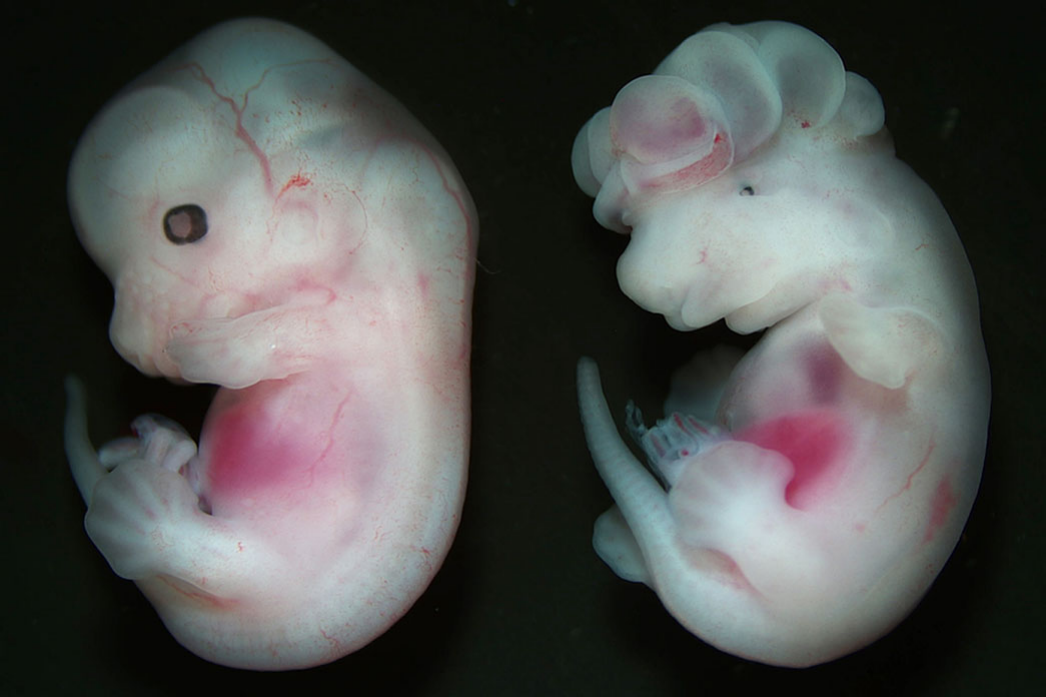
**Images of *Wdpcp^+/+^* (left) and *Wdpcp^Z11/Z11^* (right) embryos at E12.5.** The mutant embryo (right) shows craniofacial anomality, edema and polydactyly.

**Why did you choose DMM for your paper?**

We chose Disease Models & Mechanisms because of the reputation of this journal's publisher, The Company of Biologists, which is dedicated to both serving the biological science community and publishing high-quality papers. The quality of published papers is consistently high. We are proud to be able to make similar contributions. Pertinent to this study, DMM provides an ideal platform to connect basic biological mechanisms to human disease. By reporting a novel genetically engineered mouse model and mechanistic insight into how a mutation can lead to ciliopathies, our paper fits well with the journal's focus on disease-relevant models. DMM is also widely read by both basic researchers and clinicians, which means our findings can reach a broad audience interested in developmental biology, genetics and translational research.

**Given your current role, what challenges do you face and what changes could improve the professional lives of other scientists in this role?**

After postdoc training, I became an independent investigator. The biggest challenge I face is securing stable research funding. In Korea, the size of research grants is much smaller compared to grants in the United States, where I was trained. Recent cuts to government research and development budgets made this situation more challenging. Researchers like me struggle to sustain basic science research projects and support students who have aspirations in basic science. I believe that investment in basic research should be recognized as a long-term commitment rather than something easily influenced by short-term policy or political shifts. Steady and consistent funding is essential for building solid scientific foundations. At the same time, researchers have the responsibility to dedicate themselves fully to their work so that this investment translates into meaningful discoveries.

**What's next for you?**

My next step is to further stabilize my laboratory and expand the research I began at Stony Brook University (Chen lab). While much of my work so far has focused on primary cilia, I am now extending this to motile cilia, with the goal of understanding their functions and roles in disease. I am also trying to discover and develop drugs that can improve cilia formation and function, which may help prevent or treat ciliopathies and other related conditions. My team is screening synthetic compounds provided by our collaborator and has recently identified a promising molecule that enhances the expression and function of motile cilia, which are reduced in chronic obstructive pulmonary disease. We hope that this line of research will open new therapeutic opportunities for this progressive disorder.

**Tell us something interesting about yourself that wouldn't be on your CV**

Recently, I became a mother of two young children – a 3-year-old daughter and a 1-year-old son. Raising them has brought me a kind of joy I had never experienced before. Watching them grow and develop day by day constantly reminds me of the wonder of the human body and the dignity of life. I hope they continue to grow up healthy and live very happy lives.
